# High-Throughput Screening for Drugs That Inhibit Papain-Like Protease in SARS-CoV-2

**DOI:** 10.1177/2472555220963667

**Published:** 2020-10-10

**Authors:** Emery Smith, Meredith E. Davis-Gardner, Ruben D. Garcia-Ordonez, Tu-Trinh Nguyen, Mitchell Hull, Emily Chen, Pierre Baillargeon, Louis Scampavia, Timothy Strutzenberg, Patrick R. Griffin, Michael Farzan, Timothy P. Spicer

**Affiliations:** 1 Department of Molecular Medicine, Scripps Research Institute, Scripps Florida, Jupiter, FL, USA; 2 Immunology and Microbiology, Scripps Research Institute, Scripps Florida, Jupiter, FL, USA; 3 Calibr at Scripps Research, La Jolla, CA, USA

**Keywords:** COVID-19, HTS, PLpro, cell-based

## Abstract

The emergence of severe acute respiratory syndrome coronavirus 2 (SARS-CoV-2) in late 2019 has triggered an ongoing global pandemic whereby infection may result in a lethal severe pneumonia-like disease designated as coronavirus disease 2019 (COVID-19). To date, millions of confirmed cases and hundreds of thousands of deaths have been reported worldwide, and there are currently no medical countermeasures available to prevent or treat the disease. The purported development of a vaccine could require at least 1–4 years, while the typical timeline from hit finding to drug registration of an antiviral is >10 years. Thus, repositioning of known drugs can significantly accelerate the development and deployment of therapies for COVID-19. To identify therapeutics that can be repurposed as SARS-CoV-2 antivirals, we developed and initiated a high-throughput cell-based screen that incorporates the essential viral papain-like protease (PLpro) and its peptide cleavage site into a luciferase complementation assay to evaluate the efficacy of known drugs encompassing approximately 15,000 clinical-stage or US Food and Drug Administration (FDA)-approved small molecules. Confirmed inhibitors were also tested to determine their cytotoxic properties. Here, we report the identification of four clinically relevant drugs that exhibit selective inhibition of the SARS-CoV-2 viral PLpro.

## Introduction

The current pandemic of COVID-19 (coronavirus disease 2019) caused by a novel coronavirus, severe acute respiratory syndrome coronavirus 2 (SARS-CoV-2), has led to more than 30 million confirmed cases and approximately 1,000,000 deaths in more than 100 countries (as of September 29, 2020, per the World Health Organization) since its emergence in late 2019. Many patients infected with the virus develop acute respiratory distress syndrome (ARDS), which may lead to long-term reduction in lung function, arrhythmia, and death. Many approaches are being taken to develop a vaccine against SARS-CoV-2; however, it may be years until an effective vaccine is deployable to a sufficient portion of the population for herd immunity. Until a vaccine is available, the main tools for addressing the pandemic are mitigation or suppression techniques aimed at limiting the spread of infection within the population. These focus on social distancing, especially in elderly and high-risk populations; widespread use of face masks; and extensive testing, contact tracing, and isolation programs to identify all infected individuals. Antiviral therapeutics, using known and approved drugs, can be a faster method to decrease the severity of the viral infection while efforts to create a vaccine are underway.

Coronaviruses encode multiple enzymes that are essential to replication. These include two proteases, main protease [Mpro; also called 3-chymotrypsin-like protease (3CLpro)] and papain-like protease (PLpro); a replication complex comprising an RNA-dependent RNA polymerase complex (RdRp) consisting of nsp7, nsp8, and nsp12; an endonuclease, nsp15; and a number of other accessory proteins.^1^ Studies have sought to inhibit coronavirus replication by inhibiting each of these enzymes, but the drugs that are closest to market are nucleoside analogs that inhibit the replicase complex, such as remdesivir, GRL0617, and EIDD-1931.^2–5^ These drugs are, however, effective only if administered early during the course of infection.^1,6,7^

**Figure 1.**
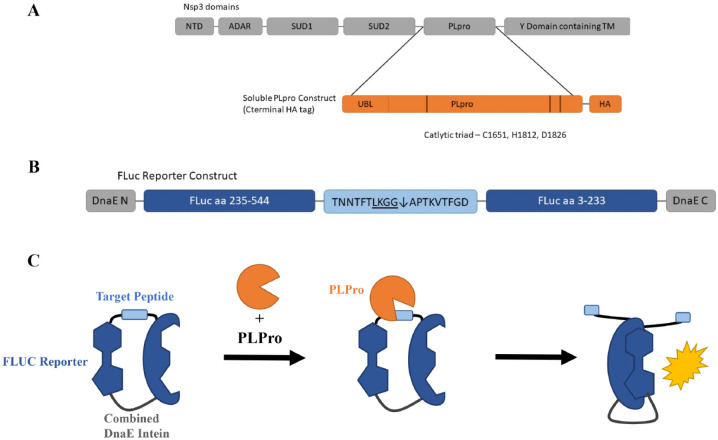
Schematic of assay design. (**A**) Schematic of the multidomain Nsp3 protein containing papain-like protease (PLpro). Inset details the soluble PLpro construct used in this study, consisting of the ubiquitin-like (Ubl) domains, the PLpro catalytic core, with a C-terminal hemagglutinin (HA) tag. (**B**) Schematic of the firefly luciferase (FLuc) reporter construct. C- and N-terminal portions of a FLuc gene are separated by a target peptide containing the cleavage sequence from the junction of nsp2 and nsp3. N- and C-terminal DnaE inteins assist in dimerization once cleaved. (**C**) Schematic of the assay. In the presence of PLpro, the target peptide is cleaved, and FLuc domains dimerize and are catalytically active.

The two proteases, Mpro and PLpro, are also attractive targets for antiviral drugs. These enzymes are essential for processing the polyproteins that are translated from the viral RNA. Each protease cleaves at different, specific sites in the polyprotein, with Mpro cleaving at 11 sites and PLpro cleaving at three sites. Inhibitors of these essential proteases would limit viral replication and therefore limit infection in patients. Although Mpro cleaves more viral sites, PLpro has additional targets, which makes it a potentially important drug target. PLpro resides in the multidomain Nsp3 protein and is responsible for the cleavages of the N-terminus of replicase polyprotein 1a to release Nsp1, Nsp2, and Nsp3. In addition, PLpros of SARS-CoV-1 and other coronaviruses are known to limit the innate immune response to infection by cleaving ubiquitin and ISG15 (ubiquitin-like interferon-stimulated gene 15) from key signaling molecules, thereby preventing the production of interferon and inhibiting antiviral effects.^1,3,8,9^ The most severe cases of COVID-19 involve a dysregulation of immune responses, resulting in a dampened interferon response and enhanced inflammatory responses. Therefore, preventing PLpro from disrupting immune signaling may ameliorate these effects. These characteristics make PLpro a desirable target for treating coronavirus infections, but no inhibitor has been approved by the US Food and Drug Administration (FDA) for marketing. Here, we aim to find drugs that inhibit the PLpro enzyme. This was done by using transient transfected cells with PLpro and a luciferase-based reporter. Inhibitors of PLpro will decrease the luminescence as compared to untreated cells. These inhibitors were further tested for their inhibition of PLpro in enzymatic and other cell-based assays to determine their direct effect on the enzyme. The high-throughput assay design can be found in **Figure 1**.

We have implemented this assay principle to run a 1536-well high-throughput assay, testing approximately 15,000 compounds inclusive of three libraries of compounds that either have some prior clinical testing or were molecularly docked versus the PLpro enzyme. At the completion of the campaign, we found four compounds that had partial selectivity to inhibiting the PLpro enzyme. These four compounds were subjected to various secondary assays to show mode of action. The results of the high-throughput screen and secondary assays are found here.

## Materials and Methods

### Plasmids

Firefly luciferase (FLuc) reporters were designed to respond on cleavage of a target peptide based on previously described picornavirus protease reporters.^10,11^ A codon-optimized gene fragment encoding the DnaE intein, C-terminal FLuc fragment, cleavage peptide, and N-terminal FLuc fragment was cloned into the CMVR expression vector between NotI and BamHI restriction sites. The cleavage peptide sequence (amino acids TLKGG↓APTKV) was derived from the native sequence of the nsp2–nsp3 junction from the Wuhan-IVDC-HB-01-2019 EPI_ISL_402119 isolate. The coding sequence for soluble PLpro with a C-terminal hemagglutinin (HA) tag was synthesized and cloned into the CMVR expression vector from the same reference sequence. Analogous constructs were synthesized for SARS-CoV-1 PLpro (Urbani strain, accession AY278741_1, peptide: RLKGG↓APIKG) and the HIV protease (matrix/capsid peptide: VSQNY↓PIVQ) as controls. Plasmids were amplified and purified from DH5α *Escherichia coli* using Invitrogen Maxiprep kits (Invitrogen, Waltham, MA) and fully sequenced to confirm the correct sequence.

### MaxCyte Transient Transfection

The MaxCyte transfection system was chosen over lipid-based methods due to its superior scalability and affordability.^12^ Briefly, 293T cells were grown in Dulbecco’s modified Eagle medium (DMEM) supplemented with 10% heat-inactivated fetal bovine serum (HI FBS) and 1% Anti-anti (all media reagents from Life Technologies, Carlsbad, CA). At 70–90% confluence, the 293T cells are harvested and resuspended in MaxCyte Electroporation Buffer at 1e8 cells/mL. DNA is added to the cells in the following ratios: 37% PLpro or empty vector for high control cells, 55% FLuc reporter–PLpro, and 9% renilla plasmid (may be used for built-in cytotoxicity analysis, but we did not). The cells are electroporated using MaxCyte cassettes and the MaxCyte device per the manufacturer’s instructions. The cells are incubated for 20 min prior to seeding in flasks for a 4 h incubation. The cells were harvested and stored in liquid nitrogen to be used during high-throughput screening (HTS).

### PLpro 1536-Well Luciferase Assay

The PLpro and empty vector cells were thawed and counted. Compounds were pre-spotted onto fresh assay plates with either 5 nL (for 10 mM stocks of ReFRAME) or 20 nL (for 1 mM or 2.5 mM stocks of Pathogen Box or Target Mol). The cells were seeded at 2500 cells/well or 5e5 cells/mL in 293T growth medium using a BioRaptr FRD (Flying Reagent Dispenser; LGR, Carlsbad, CA) at 5 µL/well. The plates were briefly spun at 1000 rpm and incubated for 48 h at 37 °C, 5% CO_2_, and 95% relative humidity (RH). After a 48 h incubation, the plates were removed from the incubator and allowed to equilibrate to room temperature for 15 min. ONE-Glo (Promega, Madison, WI) luciferase reagent was added at 5 µL/well with the BioRaptr FRD, and the plates were again briefly spun. After a 10 min incubation at room temperature, the luminescence was measured using a ViewLux (PerkinElmer, Waltham, MA) for 30 s. The high control was empty vector + FLuc wells, and the low control and data wells had PLpro + FLuc + compound or vehicle (DMSO).

### Post-HTS Confirmation Assay

Following the completion of screening all three libraries, the most active and selective drugs were subjected to testing under the following conditions. HEK293T cells were transiently transfected in 6-well plates using jetPRIME transfection reagent (Polyplus, Illkirch-Graffenstaden, France), according to the manufacturer’s instructions, at the same ratios used in the MaxCyte transfection. After 4 h, transfection complexes were removed, and cells were reseeded into 96-well plates containing compounds at a density of 20,000 cells per well. Plates were then incubated at 37 °C for 48 h. FLuc and renilla luciferase (RLuc) luminescence were detected using the Promega Dual Glo kit according to the manufacturer’s instructions. This procedure was done using both the SARS1 and SARS2 reporter systems, using plasmids with their analogous peptides based on the details referenced in the plasmid methods.

### Histidine-Tagged Small Ubiquitin-Like Modifier (His-SUMO) SARS-CoV-2 PLpro (1564–1877) Expression and Purification

As a further test of specificity, we also characterized the most potent and selective drugs using a targeted biochemical SARS2 enzyme activity assay. First, we had to produce the enzyme. The SARS-CoV-2 PLpro (1564–1877, MN908947.3) amino acid sequence was codon optimized for *E. coli* expression, subcloned, and sequence verified (GenScript, Piscataway, NJ) into the pE-SUMOpro AMP vector (LifeSensors, Malvern, PA). This vector was transformed into One Shot BL21(DE3) competent cells (Thermo Scientific, Waltham, MA) and plated onto LB-AMP plates (InvivoGen, San Diego, CA). Transformants were inoculated in 100 mL terrific broth (TB) medium supplemented with 50 µg/mL carbenicillin and incubated overnight at 37 °C with shaking to saturation (OD_600_ ≥ 2). The overnight culture (~1:50 dilution) was used to inoculate fresh TB medium supplemented with 50 µg/mL carbenicillin. A 3 L culture was incubated at 37 °C with shaking to OD_600_ ~0.4, induced by adding IPTG to a final concentration of 0.5 mM, and cultured for an additional 24 h at 20 °C, again with shaking. Cells were harvested by centrifugation, and the cell pellet was stored at −80 °C. The cell pellet was thawed on ice and resuspended in lysis buffer (50 mM HEPES, pH 8.0, 500 mM NaCl, 10 mM imidazole, 10% glycerol), DNase I (5 µg/mL), and 1× SigmaFast protease inhibitor (Sigma Aldrich, St. Louis, MO) at 5 mL per gram of cell pellet weight. Cells were lysed using a French Press (Avestin, Ottawa, Canada) following the manufacturer’s instructions. Cell lysate was centrifuged at ~51,000 × g for 60 min at 4 °C. Using an ÄKTA Pure purifier system (Cytiva, Marlborough, MA) maintained at 4 °C, the clear supernatant was loaded into a Ni-NTA Superflow column (Qiagen, Hilden, Germany) previously equilibrated with 10 column volumes of Ni-Buffer A (50 mM HEPES, pH 8.0, 500 mM NaCl, 10 mM imidazole, 10% glycerol). The column was washed with the same buffer until the A_280_ returned to its baseline value. Bound protein was eluted with a linear gradient of Ni-Buffer B (50 mM HEPES, pH 8.0, 500 mM NaCl, 250 mM imidazole, 10% glycerol), and 1 mL fractions were collected. Samples of eluted protein were analyzed by sodium dodecyl sulfate–polyacrylamide gel electrophoresis (SDS-PAGE) gel (BioRad, Hercules, CA) and Odyssey Western Blot system probed with anti-SUMO-tag Rabbit Anti-Smt3 antibody (Abcam, Cambridge, UK). Confirmed fractions were pooled and concentrated, and their buffer was exchanged with storage buffer (50 mM HEPES, pH 8.0, 500 mM NaCl, 10% glycerol) to near 190 µM using Amicon Ultra-15 10 kDa units (Thermo Scientific). Final concentration was ~9.0 mg/mL, and purity estimated at >80%.

### Fluorescence-Based SARS-CoV-2 PLpro Enzymatic Assay

To determine the IC_50_ values for the lead inhibitors in the SARS2 target-based assay, a modified inhibition assay (21 µL), from the previously published method,^13^ was performed in triplicate in a 384-well plate format. After protein, peptide, and buffer optimizations, the final His-SUMO SARS-CoV-2 PLpro (1564−1877) enzyme concentration was 0.15 µM. The assay was performed at 25 °C using HEPES (pH 7.4) as the assay buffer, and the enzyme activity was monitored by measuring the PLpro-mediated release of 7-amino-4-methylcoumarin (AMC) from the 25 µM ZRLRGG-AMC peptide substrate (Bachem, Bubendorf, Switzerland), using the EnVision multimode plate reader (PerkinElmer) with Umbelliferone filters (excitation wavelength of 360 nm with a 40 nm bandwidth, and emission wavelength of 460 nm with a 50 nm bandwidth). Briefly, using a HiBase black polystyrene 30 µL 384-well plate (Greiner Bio-One), 7 µL of 3× concentration His-SUMO SARS-CoV-2 PLpro (1564−1877) enzyme and 7 µL of 3× concentration inhibitor (final 12 points 1:3 dose response starting at 30 µM) were mixed and incubated for 30 min at 25 °C before adding/mixing 7 µL of 25 µM ZRLRGG-AMC peptide. All reagents were dissolved in assay buffer. Plates were incubated for 30 min at 25 °C before fluorescence was measured.

Following reader acquisition, we normalized all data using the average raw data per test drug concentration minus the average raw of the DMSO control divided by the average raw data for the highest concentration of disulfiram minus the average raw of DMSO*100. IC_50_s were derived using an unconstrained four-parameter fit with GraphPad Prism.

In this assay, we validated experimental performance using recently identified inhibitors of PLpro enzyme activity, disulfiram and GRL-0617, with anticipated IC_50_s of 2 µM and 0.7 µM, respectively.

### Screening Libraries

#### ReFRAME Library

Calibr has partnered with the Bill and Melinda Gates Foundation to form an integrated platform of drug candidates, the ReFRAME library. ReFRAME contains greater than 13,000 purchased or resynthesized FDA-approved/registered drugs (~40%), as well as investigational new drugs currently or previously in any phase of clinical development (~60%).

#### Pathogen Box Library

The Pathogen Box library is 400 diverse, drug-like molecules active against neglected diseases of interest. It is provided by the Medicines for Malaria Venture.

#### TargetMol Library

TargetMol performed a computer-aided drug design (CADD) in silico docking using the Swiss-Model Homology Modeling process to generate reliable protein models or 3D protein structures of receptor binding domain (RBD) of spike protein, ACE2, viral PLpro, and Mpro. Based on the protein structure of PLpro, TargetMol selected 474 top-ranked docked molecules into the PLpro-targeted compound library (CADD) by molecular docking and virtually screening against 15,376 compound structures.

#### Screening Data Acquisition, Normalization, Representation, and Analysis

All data files were uploaded into the Scripps institutional HTS database (Symyx Technologies, Santa Clara, CA) for plate quality control (QC) and hit identification. Activity for each well was normalized on a per-plate basis using the following equation:

(1) $$\%activity=100\times \left(\frac{\begin{array}{c} Test\;well-\\ Median\;Low\;\;Control \end{array}}{\begin{array}{l} Median\;High\;Control-\\ Median\;Low\;\;Control \end{array}}\right)$$

where “High Control” represents wells containing cells transfected with empty vector + FLuc; “Low Control” represents wells containing DMSO and cells transfected with PLpro + FLuc; and the “Data Wells” contain the same with test compounds. The Z’ and signal-to-background ratio (S:B) were calculated using the high control and low control wells. In each case,^19^ a Z’ value greater than 0.5 was required for a plate to be considered acceptable.^14,23^

## Results

### 1536-Well-Format Assay Optimization

**Figure 2.**
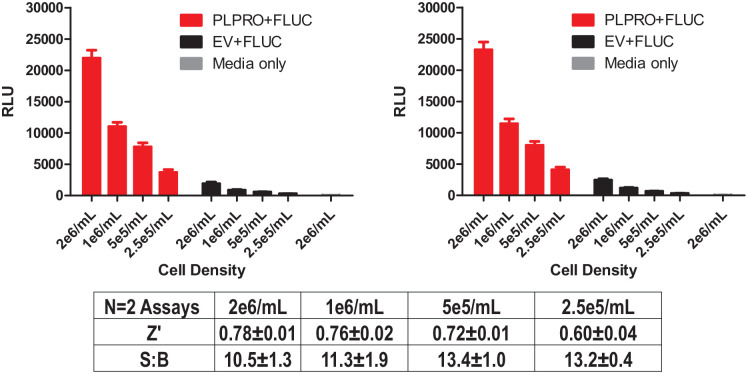
Optimization of cell-seeding density. In this 1536-well format, a range of seeding concentrations were tested to determine the lowest concentration of cells per well that yielded an acceptable Z’. Comparison of papain-like protease (PLpro) firefly luciferase (FLuc) (shown in red) versus enterovirus (EV) FLuc (black) allowed us to select the cell number that afforded the best condition for the high-throughput screening (HTS) effort (see text). The medium-only control (gray) was used to demonstrate no activity would be found when cells aren’t present (i.e., background).

Due to the assay’s excellent performance, with Z’s consistently greater than 0.5, only limited optimization was done. Changes were limited to the transfection method, creating a freezer-ready assay and a cell titer at seeding for the assay. Assay optimization was done to scale the transfection for large-scale production of transiently transfected cells that were frozen in bulk for later use in the 1536-well assay. A head-to-head comparison of the JetPRIME system to MaxCyte was done, and results with MaxCyte were similar, if not better. Fresh versus frozen transient cultured cells were also compared again with non-deleterious effect on Z’s. Finally, we optimized the cell-seeding density to understand the assay sensitivity to changes in cell number per well. Since there is no small-molecule control for this assay, we predisposed our results toward excellent Z’s (always >0.5) while limiting the cell number to be as low as possible, which we perceived would allow for more sensitivity to identify weak inhibitors. The outcome of cells seeded at varying densities at 5 µL/well in a 1536-well format is shown in **Figure 2**. Based on Z’ analysis using the controls and criteria as defined above, we chose to proceed with 5e5 cells/mL, or 2500 cells per well.

### Primary HTS

The first step of the HTS campaign was primary screening of the PLpro inhibitor against the ReFRAME library.^15^ In this primary screen, 13,104 compounds were tested at a single concentration in singlicate at a final nominal concentration of 10 µM. Raw assay data were imported into Scripps’ corporate database and subsequently analyzed using Symyx software. Activity of each compound was calculated on a per-plate basis using Equation (1).

**Figure 3.**
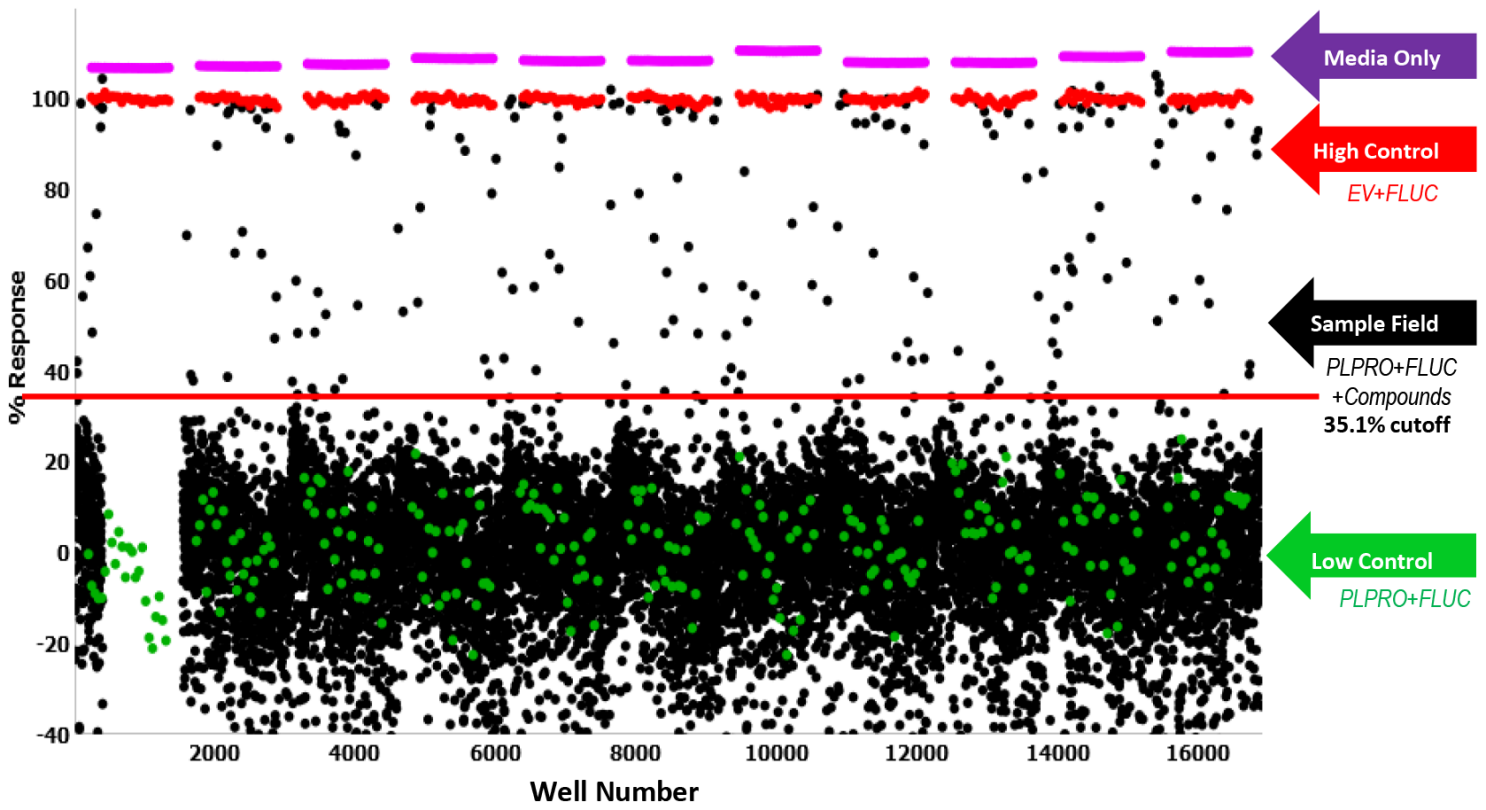
Papain-like protease (PLpro) ReFRAME primary high-throughput screening (HTS) assay results. Graphed is a single-point scatterplot of all 13,104 compounds tested. Each dot graphed represents the activity result of a well containing test compound (black dots) or controls (red, violet, and green dots). The gap in the sample index (well number) is due to test wells that weren’t inclusive of compounds, and hence no data are plotted and available.

Assay performance was excellent, with an average Z’ of 0.71±0.04 and an average S:B of 11.38±1.57 (*n* = 11 plates). A summary of the results of the primary screening assay are shown in **Figure 3** and **Table 1**. A mathematical algorithm was used to determine active compounds. Here we applied what we call a DMSO cutoff. In this case, the average activity of all DMSO-treated wells from multiple interleaved DMSO-only plates, plus three times the standard deviation value for the same set of data, was used as a cutoff parameter (i.e., any compound that exhibited greater percent activation than the cutoff parameter was declared active). Using this DMSO cutoff criteria of 35.1%, the primary assay yielded 212 active compounds (“hits”), all of which were picked for confirmation along with several new antiviral compounds for a total of 235 compounds for confirmation and counterscreen.

**Table 1.** HTS Campaign Statistics.

Library	Stage	Concentration	# Samples	# Replicates	# Plates	Z’	S:B	Hit Cutoff	# Hits
**ReFRAME**	Primary	10 µM	13,104	1	11	0.71±0.04	11.38±1.57	35.1%	212
	Confirmation	10 µM	235	3	1	0.75	15.48	27.5%	210
	Titration	20 µM	210	3	6	0.72±0.03	18.39±1.30	IC_50_ < 10 µM	164
	Titration CS	20 µM	210	3	6	0.76±0.02	3.02±0.15	IC_50_ < 10 µM	185
**Pathogen Box**	Primary	4 µM	398	3	3	0.70±0.02	10.28±0.47	28.0%	16
	Confirmation	4 µM	12	3	3	0.72±0.02	10.98±0.98	27.3%	3
	Counterscreen	4 µM	12	3	3	0.68±0.04	2.58±0.19	21.3%	8
**Target Mol**	Confirmation	4 µM	1097	3	6	0.75±0.05	17.23±1.46	34.3%	27
	Counterscreen	4 µM	1097	3	6	0.75±0.04	3.04±0.04	10.10%	132
	Titration	10 µM	27	3	3	0.71±0.09	11.27±0.86	IC_50_ < 4 µM	19
	Titration CS*	10 µM	27	3	3	0.86±0.01	28.61±1.12	IC_50_ < 4 µM	24

CS: counterscreen; S:B: signal-to-background ratio.

### Secondary Assay

After completion of cherry-picking and pre-spotting, the confirmation PLpro used the same reagents and detection system as the primary screening assay but tested each of the 235 compounds at a single concentration (nominally 10 µM) in triplicate. Performance of the PLpro HTS confirmation assay was consistent with previous experiments, with an average Z’ of 0.75 and an average S:B of 15.48. Again, we ascertained a DMSO cutoff, and in this case 27.5% activity was used to obtain 210 hits or an 89% hit rate. A summary of the results of the confirmation is in **Table 1**. All 210 active compounds were selected for titration assays.

### Titration Assay

After completion of cherry-picking and pre-spotting, the titration PLpro assay used the same reagents and detection system as the primary and secondary HTS assays, but tested each of the 210 compounds at a starting concentration of 20 µM, nominally in triplicate, with a 1:3 dilution series. Assay performance of the PLpro inhibitor was good, with an average Z’ of 0.72±0.03 and an average S:B of 18.39±1.30 (*n* = 6 plates). For each test compound, percent activation was plotted against compound concentration. A four-parameter equation describing a sigmoidal dose–response curve was then fitted with an adjustable baseline using Assay Explorer software (Symyx Technologies). The reported IC_50_ values were generated from fitted curves by solving for the X-intercept value at the 50% activation level of the Y-intercept value. The following rule was used to declare a compound as “active” or “inactive”: Compounds with an IC_50_ >10 µM were considered inactive. Compounds with an IC_50_ ≤10 µM were considered active. Of those, 164 compounds were active in the PLpro assay.

### Cytotoxicity Titration Counterscreen

The counterscreen titration assay used the same PLpro + FLuc cells and protocol as the primary assay but used CellTiter-Glo (Promega) to determine the effect of drug cytotoxicity on the cells. The low control and sample field were set up under the same exact conditions as the primary screen. The high control, in this case, was determined as the PLpro + FLuc cells with the addition of 10 µM doxorubicin, which is a common drug that shows toxicity in cell lines, as was the case here as well, demonstrating complete or near-complete toxicity. Assay performance of the PLpro inhibitor cytotoxicity assay was good, with an average Z’ of 0.76±0.02 and an average S:B of 3.02±0.15 (*n* = 6 plates). 185 compounds showed IC_50_s <10 µM. The data from the PLpro and the cytotoxicity counterscreen can be seen in **Table 1**. A simple two-way Venn overlap was done to understand the relationship of active molecules in either assay, which elucidated only four hits with moderate selectivity over the cytotoxicity counterscreen. These drugs, along with their associated IC_50_s for PLpro and the cytotoxicity assay, respectively, are: bleomycin (0.7 µM and 7.7 µM), R-547 (0.7 µM and 1.9 µM), ripasudil (15 µM and >20 µM), and rho-kinase-IN-1 (15.2 µM and >20 µM). All IC_50_ data have been deposited in the ReFRAME database at https://reframedb.org/assays.

### Alternative Libraries Tested

To expand on the limited number of potentially selective hits identified above, we sought to test other libraries that would allow us to diversify our panel of test inhibitors using drugs or drug-like molecules that had been molecularly docked to the PLpro target or were potentially useful as known pathogen inhibitors. Thus, we tested both the Target Mol and Pathogen Box libraries, which were also screened in the same formats as the ReFRAME library. The data from these libraries can be found in **Table 1**. Neither the Target Mol nor the Pathogen Box identified any hits that were selective, and hence these weren’t pursued further.

### Downstream Characterization

**Figure 4.**
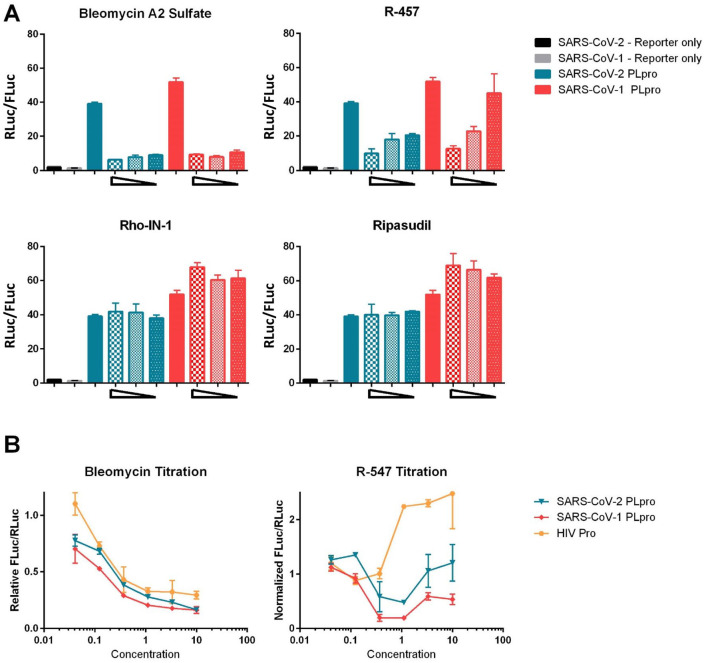
Confirmation of ReFRAME hit compounds in luciferase assay. Luciferase-based protease assays were performed in 96-well plates, as described in **Figure 1**. Cells were transfected in 6-well plates and then reseeded into 96-well plates containing compounds at 20,000 cells per well for 4 h post transfection. (**A**) Severe acute respiratory syndrome coronavirus 2 (SARS-CoV-2) papain-like protease (PLpro) and SARS-CoV-1 PLpro activity in the presence of compounds at threefold dilutions starting at a maximum concentration of 20 µM. (**B**) Activity of indicated proteases represented as relative to protease without compound treatment. Compounds were added at threefold dilutions starting at 10 µM.

Ultimately, due to overlapping cytotoxicity found in the Target Mol and Pathogen Box libraries, only the four ReFRAME library drugs that appeared to be nontoxic and active in the PLpro assays were tested for additional characterization: bleomycin, R-547, ripasudil, and rho-kinase-IN-1. First, we replicated the screen conditions in a low-throughput 96-well plate assay. The PLpro of SARS-CoV-1, which is 83% identical to that of SARS-CoV-2, was included to determine if inhibitors would be cross-reactive. In this assay, we reproduced the results of the screen for both bleomycin and R-547, and showed that they also have an effect on the activity of SARS-CoV-1 PLpro. Conversely, neither rho-kinase-IN-1 nor ripasudil had any inhibitory effect in these conditions (**Fig. 4A**). We further tested a wider range of concentrations of bleomycin and R-547, and included the unrelated HIV protease as a negative control. Surprisingly, bleomycin inhibited all three proteases to similar levels, which appeared to be primarily a result of cytotoxicity. R-547, however, did show some specificity for the coronavirus (CoV) proteases. It also had cytotoxic effects at higher concentrations, indicated by the upward slope of the normalized FLuc/RLuc ratio (**Fig. 4B**). The range between activity and cytotoxicity is, however, very narrow and requires further investigation.

**Figure 5.**
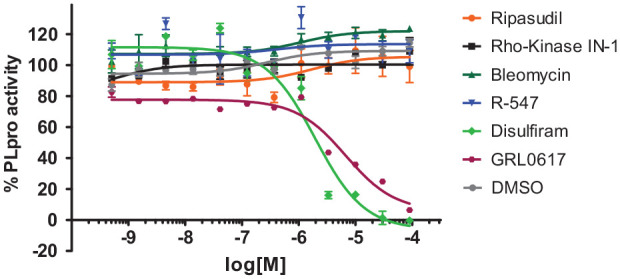
In vitro papain-like protease (PLpro) inhibition assay. 7-Amino-4-methylcoumarin (AMC) release from ubiquitin–AMC in different concentrations of test inhibitors disulfiram or GRL-0617 was measured and normalized against DMSO control. The IC_50_s of GRL-0617 and disulfiram were consistent at 6 µM and 2 µM, respectively. DMSO (vehicle only) was included as a null control. Data are presented as mean ± standard deviation (SD; *n* = 3) for an *N* of two separate experiments.

Next, we tested each of the four ReFRAME hits in the target-based biochemical SARS2 fluorescence-based assay. Each drug was tested across a full concentration response profile and yielded no activity. Preliminary unpublished reports have demonstrated IC_50_s for disulfiram or GRL-0617 as inhibitors of PLpro enzymatic activity, with values of ~6–7 µM and 0.7 µM, respectively. Our experimental value for disulfiram is 2 µM, and it is 6 µM for GRL-0617, which gives us confidence we are within a reasonable range of sensitivity for this assay. Each experiment was reproduced at least two times with at least three replicate points per concentration tested (**Fig. 5**).

## Discussion

The COVID-19 pandemic is an immediate threat, and treatments are needed quickly, so we rapidly screened three libraries of compounds that either have some prior clinical testing or were molecularly docked versus the PLpro enzyme. The premise was that this will allow any hits in this research to be quickly advanced and be available more readily for clinical translation. The HTS campaign led to a few possible PLpro inhibitor leads, which were advanced to enzymatic and other secondary studies. All said, four compounds were found to have moderate potency in respect to the cytotoxicity counterscreen. We performed both SARS1 and SARS2 cell-based PLpro follow-up studies and enzyme inhibition assays on the four compounds we thought looked interesting based on a moderate level of preferential activity in the primary assay versus the cytotoxicity profile: bleomycin, ripasudil, rho-kinase-IN-1, and R-547. Two of these compounds, bleomycin and R-547, had confirmed activity against both SARS-CoV-2 and SARS-CoV-1 PLpro in a low-throughput version of the initial assay. Further titration of these compounds indicates that the cytotoxicity is responsible for the effect of bleomycin, however, and that the inhibitory range of R-547 limits further utility.

Since we began this research, multiple groups have conducted similar screens for compounds that inhibit one or both of the SARS-CoV-2 proteases. Many of these screens have used a cell-free system involving purified enzyme and Förster resonance energy transfer (FRET)-based reporters.^16^ While these studies have identified different potential inhibitors, they have a crucial drawback: cytotoxicity. By beginning our screen with a cell-based system, we sought to identify compounds that inhibit PLpro but cause minimal cytotoxicity. This is an important added value to our study because it eliminates compounds with toxic activities early in the screening process. Using the cell-based system also allows elimination of compounds that have poor cell permeability, because such compounds would be unlikely to have high therapeutic potential. When using cell-based systems, however, it is possible to identify hits that modify the cellular machinery instead of directly targeting the enzyme in question. To exclude this possibility, we tested the hits of our initial screen using the fluorogenic peptide assay (**Fig. 5**).

Further downstream characterization of hit compounds should include an investigation into their activity on the deubiquitinating (DUB) and deISGylation activity of PLpro, because this is a key activity that overlaps partially, but not completely, with proteolytic activity for CoV PLpro.^17–19^ Indeed, some recent studies have focused primarily on targeting this activity to modulate the immune evasion of SARS-CoV-2.^20–22^ In addition, because PLpro resides in a multidomain, membrane-bound protein, it is possible that compounds that inhibit the soluble protease may not have as great of an effect on the full nsp3 protein and therefore on viral replication.^23^ In the studies in Refs. ^20–22^ focused on the DUB activity, two compounds, GRL-0617 and 6-thioguanine, were able to inhibit SARS-CoV-2 replication in Vero E6 cells, while the third was found to be a nonspecific inhibitor of PLpro. GRL-0617 has also been confirmed to limit the innate immune evasion by SARS-CoV-2.^8^ While our current study did not identify any compounds that warranted testing for antiviral activity, this is the most important follow-up to any inhibitor screen.

While we were successful at rapidly screening large libraries of drugs that may be repurposed, this target has proven to be refractory to identifying any bona fide leads. This was somewhat surprising considering the robustness of the cell- and biochemical-based approaches and the considerable reproducibility of the hits from the vast diversity of drugs found in each library. Ultimately, the cytotoxicity of the drugs against the same cells under the same conditions proved to be an excellent counterscreen to remove liabilities early on. It clearly limited the pool of drugs for further follow-up, however. Ultimately, we have been limited in terms of pharmacologic controls to implement that would allow us to gauge the sensitivity of the cell-based and even biochemical assays. In fact, at the beginning of this effort, no known SARS2 PLpro inhibitors existed. Subsequently, two new reports (currently under peer review) alluded to disulfiram and GL-0617 as possible inhibitors. These are still being validated but may be useful to gauge our assay going forward. As bona fide inhibitors of PLpro become more widely available, we may be able to implement them for future validation of our HTS assay to help find the proper sensitivity. The biochemical approach developed as part of the downstream validation may prove to be the better path forward to identify hits and then triage with the arsenal of well-poised cell-based assays we have in place.

We have successfully completed an HTS campaign, identifying potential inhibitors of the PLpro enzyme. We have shown that we can identify compounds that inhibit the enzymatic activity of the PLpro enzyme. Future work now includes preparing for larger library screening against this target as well as others, such as SARS2 entry and Mpro.
